# Extracellular Vesicles Released by Genetically Modified Macrophages Activate Autophagy and Produce Potent Neuroprotection in Mouse Model of Lysosomal Storage Disorder, Batten Disease

**DOI:** 10.3390/cells12111497

**Published:** 2023-05-29

**Authors:** Nazira El-Hage, Matthew J. Haney, Yuling Zhao, Myosotys Rodriguez, Zhanhong Wu, Mori Liu, Carson J. Swain, Hong Yuan, Elena V. Batrakova

**Affiliations:** 1 Department of Immunology and Nanomedicine, Herbert Wertheim College of Medicine, Florida International University, Miami, FL 33199, USA; nelhage@fiu.edu (N.E.-H.); myrodrig@fiu.edu (M.R.); 2 Center for Nanotechnology in Drug Delivery, University of North Carolina at Chapel Hill, Chapel Hill, NC 27599, USA; mjhaney@email.unc.edu (M.J.H.); yulingz@email.unc.edu (Y.Z.); 3 Eshelman School of Pharmacy, University of North Carolina at Chapel Hill, Chapel Hill, NC 27599, USA; mori6@ad.unc.edu (M.L.); cjswain@ad.unc.edu (C.J.S.); 4 Department of Radiology, School of Medicine, University of North Carolina at Chapel Hill, Chapel Hill, NC 27514, USA; zhhwu@ad.unc.edu (Z.W.); yuanh@ad.unc.edu (H.Y.)

**Keywords:** autophagy, Batten disease, drug delivery, extracellular vesicles, lysosomal storage disorders, neuroprotection

## Abstract

Over the recent decades, the use of extracellular vesicles (EVs) has attracted considerable attention. Herein, we report the development of a novel EV-based drug delivery system for the transport of the lysosomal enzyme tripeptidyl peptidase-1 (TPP1) to treat Batten disease (BD). Endogenous loading of macrophage-derived EVs was achieved through transfection of parent cells with TPP1-encoding *p*DNA. More than 20% ID/g was detected in the brain following a single intrathecal injection of EVs in a mouse model of BD, ceroid lipofuscinosis neuronal type 2 (CLN2) mice. Furthermore, the cumulative effect of EVs repetitive administrations in the brain was demonstrated. TPP1-loaded EVs (EV-TPP1) produced potent therapeutic effects, resulting in efficient elimination of lipofuscin aggregates in lysosomes, decreased inflammation, and improved neuronal survival in CLN2 mice. In terms of mechanism, EV-TPP1 treatments caused significant activation of the autophagy pathway, including altered expression of the autophagy-related proteins LC3 and P62, in the CLN2 mouse brain. We hypothesized that along with TPP1 delivery to the brain, EV-based formulations can enhance host cellular homeostasis, causing degradation of lipofuscin aggregates through the autophagy–lysosomal pathway. Overall, continued research into new and effective therapies for BD is crucial for improving the lives of those affected by this condition.

## 1. Introduction

Lysosomal storage disorders (LSDs) are a group of inherited genetic disorders characterized by the accumulation of various substances within lysosomes that build up due to a deficiency in lysosomal enzymes. LSDs manifested in a range of symptoms, including developmental delays, organ damage, and neurological problems. There are over 50 different LSDs, each of which is caused by a mutation in a specific gene that encodes for a specific lysosomal enzyme. Batten disease (BD), also known as neuronal ceroid lipofuscinosis (NCL), is a group of rare and severe neurodegenerative disorders that affect children. It is characterized by the accumulation of harmful substances called lipofuscins within cells, particularly in the brain, leading to the progressive loss of nerve cells, blindness, and the development of neurological problems. Specifically, late infantile neuronal ceroid lipofuscinosis type 2 (CLN2) is one of the most common forms of LSDs. CLN2 results from a deficiency of the lysosomal enzyme tripeptidyl peptidase 1 (TPP1), a serine protease that cleaves tripeptides from the N-terminus of proteins and localizes to lysosomes and extracellularly. Without functional TPP1, there is no appropriate clearance of lipofuscin aggregates, resulting in their progressive accumulation in lysosomes. Although these aggregates buildup in lysosomes of all cells, the main damage occurs in neurons of the central nervous system (CNS) and the retina, which undergo progressive degeneration [1]. Subsequently, many tissues and organ systems are affected, with early loss of neurological functions and vision [2], leading to severe disability and early death. As such, efficient delivery of functional TPP1 to the CNS is of great importance.

The treatment of BD is challenging due to the nature of the disorder and the lack of effective therapies that can reach lysosomes in the CNS. Currently, there is no cure for BD, and treatment is largely supportive, focusing on managing symptoms and providing supportive care. This may include the use of medications to control seizures, physical and occupational therapy to improve mobility and function, and the use of assistive devices, such as wheelchairs and feeding tubes. Enzyme replacement therapy (ERT) is the most developed approach for treatment of CLN2 that is already implemented in clinics; however, successful treatment of patients can be accomplished only through catheters implanted in the lateral ventricle (intracerebroventricular) [3]. In general, the patients showed positive effects of ERT with substantial delay of motor, language and visual decline and reduction in cortical volume loss. Unfortunately, the therapeutic efficacy diminished over time [4]. Gene therapy via modified adeno-associated viruses (AAVs) is another experimental approach that is being explored as a potential treatment for BD [5,6]. This involves the introduction of a functioning copy of the mutated *tpp1* gene into cells, with the goal of restoring normal function. While gene therapy holds promise as a treatment for BD, it is still in the early stages of development and has not yet been approved for use in humans. 

The challenges in the treatment of BD highlight the need for continued research into new and effective therapies for this devastating disorder. Advances in nanotechnology research may lead to the development of new treatments that can improve the lives of those affected by this condition. Overall, many potent therapeutics require efficient delivery systems, such as nanocarriers that protect the incorporated drug against degradation, improve its pharmacokinetics, and decrease side effects. A multitude of synthetic nanocarriers, including inorganic nanoparticles [7], polymer-based nanoparticles and micelles [8,9,10], liposomes [11,12,13,14], and solid lipid-based nanoparticles (SLN) [15,16], have been developed from a variety of materials for improved drug delivery and even introduced to the market [17]. Some of them, especially SLN, have shown promising results [18,19,20,21]. However, the efficacy of conventional nanoparticles and nanoformulations is limited due to their cytotoxicity and fast clearance by phagocytes, as well as inadequate drug-loading efficiency, leakage during storage, and costly production process [22]. In addition, they have a limited ability to penetrate across biological barriers, such as the blood–brain barrier (BBB) or blood–retina barrier (BRB). As such, there is an urgent need for more biocompatible therapeutic approaches based on natural drug carriers’ systems.

EVs, exosomes, and microvesicles are small, membrane-bound vesicles that are secreted by cells. They contain a variety of biomolecules, including nucleic acids, proteins, and lipids, and are emerging as potential drug delivery vehicles. EVs are prominent nanocarriers that play important roles in the intercellular communications and can provide crucial benefits for drug delivery. EVs are small and can cross the BBB, which makes them well-suited for targeting specific areas of the brain. Unlike synthetic nanocarriers, EVs have a biological origin, making them safer and less immunogenic [23]. While conventional nanoparticles are usually cleared quickly by the mononuclear phagocyte system (MPS), EVs contain certain proteins on their surface that allow them to avoid entrapment in MPS and facilitate delivery of their therapeutic cargo to the disease site [24,25]. They also have a natural ability to target and enter specific cells, which can help increase the efficiency of drug delivery. Furthermore, due to EVs’ natural targeting capacity and ability to penetrate the interstitia of organs, they can minimize systemic toxicity [22]. These findings supported the notion that EVs have an unrivaled efficacy compared to synthetic drug nanoformulations that can provide powerful treatment for conditions lacking effective pharmacotherapy, such as neurodegenerative disorders [26]. Regarding therapeutic interventions for LSDs, EVs have a high stability and low immunogenicity, while favoring acidic conditions for fusing; therefore, they may be especially instrumental in the delivery of therapeutic enzymes to the target organelles, lysosomes. As a result, the use of EVs not only reduces the drug dosage, but also enables the administration of drugs through less invasive routes (intravenous, intranasal, intraperitoneal, etc.), providing greater benefit in the clinical aspect.

We reported earlier on the development of formulations that contained TPP1 exogenously loaded into macrophage derived EVs [27,28]. This drug delivery system was shown to provide significant neuroprotective and anti-inflammatory effects in a BD mouse model, CLN2 mice. Herein, we report a newly developed drug delivery system revolving around EVs released by autologous macrophages that were transfected with TPP1-encoding plasmid DNA (*p*DNA). We demonstrate that TPP1 delivered to the brain by means of macrophage derived EVs caused a significant improvement in the morphology and survival of neuronal cells, along with increased expression of growth factors and decreased secretion of inflammatory molecules. 

In terms of mechanism, we show that treatment with EV-TPP1 activates natural defense systems in the CLN2 mouse brain, namely, the autophagy pathway. Autophagy is a process that occurs in cells and uses lysosomes for the degradation and recycling of cellular components. This pathway is particularly important in the context of LSDs, which are characterized by an accumulation of various substances within lysosomes, the cells’ waste disposal system. Studies have shown that many LSDs, and in particular the NCLs, are characterized by an impaired autophagy pathway [29,30]. Specifically, it was discovered that the primary storage material (i.e., intracellular material that is delivered into the lysosome for degradation and recycling) disrupts the normal role of the lysosome in the autophagy pathway, resulting in the secondary storage of autophagic debris [30]. On the other hand, activation of the autophagy pathway can mitigate the effects of LSDs by providing an alternative mechanism for the degradation and recycling of cellular components. The formation of autophagosomes, characterized by small vesicles enclosed with damaged or unnecessary cellular components fuses with lysosomes for degradation. In this way, together with the lysosomal function, the autophagy pathway can prevent the toxic effects of the accumulating compounds underlying the pathophysiology of LSDs. 

Herein, we show that successful delivery of EV-TPP1 to the CLN2 mouse brain accounted for the altered expression of the autophagy-related protein 8, ATG8, /microtubule-associated protein light chain 3 (LC3), involved in the formation of autophagosomes, and the receptor for P62 (also referred to as sequestosome 1, SQSTM1), which directs protein cargo for autophagic degradation. We hypothesized that the therapeutic action of EV-TPP1 is twofold: (i) efficient delivery of therapeutically active enzyme TPP1 to lysosomes, and (ii) activation of protein aggregates clearance through the autophagy–lysosomal pathway. Both processes may be interconnected, amplifying the overall therapeutic effect. Ongoing studies in the lab using the CLN2 mouse model are currently examining how these autophagy-related processes contribute to the pathological mechanism of the NCLs and investigating the effect of EV-TPP1 on their activation. Understanding these mechanisms may provide new treatment approaches for this debilitating disorder.

## 2. Materials and Methods

### 2.1. Plasmids and Reagents

Recombinant full-length Human TPP1 protein (lot #BIQE03, UniProtKB—O14773 (TPP1_HUMAN)) was a generous gift from BioMarin Pharmaceutical Inc. (Novato, CA, USA). Lipophilic fluorescent dye, 1,1′-Dioctadecyl-3,3,3′,3′-tetramethylindo-carbocyanine iodide (DIR), was purchased from Thermo Fisher Scientific (Waltham, MA, USA). A nuclear dye, 4′,6-diamidino-2-phenylindole dihydrochloride (DAPI), was obtained from Sigma-Aldrich (St. Louis, MO, USA). Primary Anti-NeuN antibody (ab77487, neuronal marker), anti-microtubule-associated protein 2 (MAP2) (ab5392, neuronal marker), rabbit polyclonal anti-GFAP antibody (ab7260, astrocytosis marker), and anti-ATP synthase C antibody (ab181243, lysosomal storage body marker) were purchased from Abcam (Cambridge, MA, USA). Secondary antibody, goat anti-rabbit IgG H+L Alexa Flour 488 (A-11008), was purchased from Invitrogen (Carlsbad, CA, USA). Murine macrophage colony-stimulating factor (MCSF) was purchased from Peprotech Inc. (Rocky Hill, NJ, USA). Cell culture medium and fetal bovine serum (FBS) were purchased from Gibco Life Technologies (Grand Island, NY, USA). All other chemicals were reagent grade.

### 2.2. Genetic Modification of Parent Macrophages

Macrophages were transfected using the Invitrogen Neon Electroporation system. In particular, 1 × 10^6^ cells were supplemented with 15 µg TPP1 *p*DNA and electroporation buffer, and electroporated using Neon 100 µL tips. Following electroporation, the cells (125,000 cells/well) were seeded on a 24-well plate and cultured in RPMI 1640 media with 10% FBS. No antibiotics were used, as per the manufacturer’s recommendation. Then, cell-conditioned media were collected on day one through day four post transfection; EVs were isolated, and TPP1 levels were assessed by ELISA (Thermo Scientific, EH465RB), according to the manufacturer’s protocol. Cell lysates protein concentration was determined by Pierce BCA protein assay, and the obtained amount of TPP1 was normalized by cellular protein. To visualize de novo synthesis of TPP1 in genetically modified macrophages, plasmid DNA with Myc tag was used. Following 24 h after transfection, the cells were washed, permeabilized, and stained with FITC Anti-Myc tag antibody (ab1263, Abcam, Cambridge, UK).

### 2.3. EVs Isolation

Parental macrophages were grown on 75T flasks (20 × 10^6^ cells/flask) for 1 week. Then, conditioned media were collected, and EVs were isolated using differential ultracentrifugation [31]. First, the media was cleared from the cell debris and large vesicles by sequential centrifugation at 300× *g* for 10 min, 1000× *g* for 20 min, and 10,000× *g* for 30 min, followed by filtration using 0.2 μm syringe filters. Next, the EVs were pelleted by centrifugation at 100,000× *g* for 1 h. The pelleted EVs (10^11^–10^12^ particles/flask) were washed twice with phosphate buffer solution (PBS). To avoid contamination by the FBS-derived EVs, FBS was spun at 100,000× *g* for 12 h to deplete EVs before the experiment. The conditioned media were retrieved after 72 h. The protein concentration Bradford assay was used to estimate the recovery of EVs.

### 2.4. Characterization of EVs

Isolated from genetically modified macrophages, EVs were characterized by Nanoparticle Tracking Analysis (NTA), Dynamic Light Scattering (DLS), and ELISA. First, the size, distribution, and number of particles were also examined by NTA. For this purpose, EVs were prepared at a concentration 0.01 mg/mL and evaluated using NanoSight 500, Version 2.2 (Wiltshire, UK). The data were confirmed by DLS using the ZetaPlus’ Zeta Potential Analyzer (Brookhaven Instruments, Santa Barbara, CA, USA) equipped with a 35 mW solid state laser (658 nm laser), as described in [32,33]. The ZetaView QUATT Nanoparticle Tracking Microscope PMX-420 (Particle Metrix, Inning am Ammersee, Germany) was used to trace the ZP of individual particles by tracking electrophoretic movement after electric field application. EVs were diluted to a concentration of about 1 × 10^8^–5 × 10^8^ with pre-filtered PBS. Measurements were performed at 11 positions and using the following settings: maximum area 1000, minimum area 5, minimum brightness 20. Furthermore, the purity of the obtained EVs fraction was estimated as a ratio between the number of particles (obtained by NTA) and protein concentration (obtained by the Bradford assay). This assessment indicated that there is nearly 0% protein contamination in EVs fraction (2.1 × 10^10^ particles/ug protein).

The levels of proteins constitutively expressed in EVs were identified by Western blot analysis, using the Wes^™^ Simple Western Blot device (ProteinSimple, San Jose, CA, USA). EVs were lysed with 1× RIPA buffer for 30 min at room temperature, and 200 or 40 mg/mL of protein was denatured and loaded in Wes™ multi-well plates, following the manufacturer’s instructions. Protein concentrations were determined using the BCA kit (Pierce Biotechnology, Rockford, IL, USA). For analysis of CD63, samples’ lysates de-glycosylated using PNGase F PRIME (Bulldog Bio, NZPP050) under non-denaturing conditions at the ration 1:9 v:v for 1 h prior to denaturalization. The protein bands were detected with primary antibodies described in Supplementary Table S1 and secondary Goat Anti-Rabbit HRP Conjugate (ready-to-use reagent, ProteinSimple, San Jose, CA, USA). The protein concentration for all samples was kept the same, 200 µg/well. A quantitative analysis of obtained images was carried out using Compass SW software. The TPP1/CLN2 ELISA kit was used to estimate the levels of TPP1 in the cell lysates, and EVs.

### 2.5. Labeling EVs with ^64^Cu for PET Imaging and Biodistribution Studies

To assay the distribution of EVs in the different brain areas of CLN2 mice, macrophage derived EVs were labeled with ^64^Cu, as described earlier [34]. Briefly, ^64^CuCl_2_ was dissolved in NH_4_OAc buffer (pH 5.5), then 10 µL PTSM (pyruvaldehyde-bis(N4-methyl-thiosemicarbazone, 1mg/mL in DMSO) was added. The mixture was incubated at room temperature for 10 min. After purification with C18 (waters, Sep-Pak^®®^ Plus), the solvent was removed, and ^64^Cu-PTSM was redissolved in PBS (pH 7.4). EVs were incubated with ^64^Cu-pyruvaldehyde-bis(N4-methylthiosemicarbazone) (^64^Cu-PTSM) in 1 mL serum-free medium at 37 °C for 1.5 h. The radioactively labeled EVs (^64^Cu-EVs) were washed 3 times with ice-cold PBS (pH 7.4) to purify them of non-conjugated ^64^Cu-PTSM. ^64^Cu-EVs were resuspended in saline administered into CLN2 mice (2 mo. old) via intrathecal administration (48.0 ± 2.8 µCi/2 × 10^10^ particles/50 µL/mouse, *N* = 3) and imaged using the SuperArgus PET/CT system at 1 h, 24 h, and 48 h post injection. After the 48 h post-injection PET scan, the animals were euthanized. The brain and blood were collected, and the accumulation of ^64^Cu-EVs was measured using a gamma counter (2470 Wizard, Perkin Elmer). The results were presented as a percentage of the injected dose per g of tissue (%ID/g). 

### 2.6. LINCL Mice

To study the therapeutic efficacy and mechanism of EV-based formulations of TPP1, LINCL mice with mutations of the *CLN2* gene encoding TPP1 were used [35]. The TETRA-ARMS design was used for CLN2 genotyping. Specifically, mutant: 266 bp, wild type: 493 bp, and locus: 704 bp bands were visualized to identify mutant KO and wild type mice with inner primers that bind to either the wild type (WT) or mutant (KO) sequence [27]. Animals were treated in accordance with the Principles of Animal Care outlined by the National Institutes of Health and approved by the Institutional Animal Care and Use Committee of the University of North Carolina at Chapel Hill (IACUC ID: 22-034.0).

### 2.7. In Vivo Fluorescence Imaging

To study the biodistribution of EVs in CLN2 upon multiple injections, macrophage-derived EVs were labeled with DIR (Invitrogen), which is a lipophilic, near-infrared, fluorescent cyanine dye (emission peak of 790), according to the manufacturer’s instructions. To reduce fluorescence quenching by fur and autofluorescence from a solid diet, CLN2 mice were shaved and kept on a liquid diet for 48 h prior to the imaging. CLN2 mice (*N* = 4) were injected with DIR-EVs in the combination of *i.p.* (6 × 10^11^ particles/200 µL) and *i.t* injections (1.5 × 10^11^ particles/50 µL) weekly and imaged at various time points (1 h–24 d) by an IVIS-Spectrum optical imaging system (PerkinElmer, Waltham, MA, USA). For background fluorescence level evaluation, all animals were imaged before the injections. At the endpoint of the experiment (24 d), mice were sacrificed and perfused according to the protocol, and isolated main organs (liver, lungs, spleen, kidney, and brain) were imaged by the IVIS imaging system. Quantitative analysis of the levels of fluorescence in the brain was performed using the spectral instruments imaging ADL Aura software (Biocompare, Tuscon, AZ, USA). 

### 2.8. Treatments of CLN2 Mice with EV-TPP1 Formulations

CLN2 mice (*N* = 10) aged 1 week were treated with EV-TPP1 or sham EVs through a combination of *i.p.* injections (4.5 × 10^10^ particles/150 µL/mouse, every week from day 10) and *i.t.* injections (1.5 × 10^10^ particles/50 µL/mouse, every week from month 1). CLN2 mice (*N* = 10) or wild type (WT) control mice were injected with saline (the same volume). Three months later, animals were sacrificed, and main organs were isolated and examined as described below.

### 2.9. Nissl Staining

Mice were sacrificed and perfused, and brains collected were frozen in OCT (optimal cutting temperature media). Frozen brains were cut into coronal sections at 10–15 µm thickness using a CryoStarTM NX50 Cryostat (Thermo Fisher) and then placed on a warm pad to remove excess OCT. Slides were stored at −20 °C. A subset of sliced tissues was stained with Cresyl violet acetate solution (Nissl) for the detection of Nissl body in the cytoplasm of neurons that stained purple blue. In brief, tissues were exposed to xylene and rehydrated in a graded series of ethanol at 100, 95, and 75% concentrations. Sections were exposed with Nissl staining solution for 15 min; washed in distilled water; immersed in ethanol at 75%, 95%, and 100% concentrations; and then cleared by xylene. Slides were then mounted using mounting media for visualization. A total of 3 to 5 areas were randomly selected to be examined with an inverted fluorescence microscope and a 40× objective (Zeiss, Germany) by investigators who were blinded to the experimental groups.

### 2.10. H & E Staining

Another subset of sliced tissues was stained with Hematoxylin and Eosin (H&E) for the detection of morphological changes. Tissues were exposed to xylene and rehydrated with ethanol at 100, 95, and 70% concentrations, followed by staining with hematoxylin dye for 15 min. After washing in distilled water, tissue sections were stained with eosin for 20 s and then dehydrated with gradient ethanol. Sections were exposed to xylene and then mounted using mounting media for visualization. Sections were imaged with an inverted fluorescence microscope and a 40× objective (Zeiss, Germany) by investigators who were blinded to the experimental groups.

### 2.11. Immunohistochemical Staining

A subset of sliced tissues was used for immunohistochemical labeling with antibody against the neuronal nuclei (NeuN) or microtubule-associated protein 2 (MAP2) to assess the surviving neuronal cells in the brain. To detect autophagy activation and maturation, a subset of slides were immunolabeled with antibody against LC3 (ab48394, autophagosome marker) and antibody against SQSTM1/P62 (ab155686), purchased from Abcam. Briefly, slides were fixed in 4% paraformaldehyde, permeabilized with 0.1% Triton X-100, blocked in 0.1% Triton X-100 with 1% milk/1% goat serum, and immunolabeled with primary antibodies. Immunoreactivity was visualized with secondary antibodies from Molecular Probes (Carlsbad, CA, USA). Cells were mounted with Prolong Gold antifade reagent with DAPI to label cell nuclei (Thermo Fisher). To investigate whether EV-TPP1 treatments affected protein aggregates in the brain of CLN2 mice, primary antibodies to subunit c of mitochondrial ATP synthase (ab181243, 1:500 dilution) were used for the accumulation of lysosomal storage bodies and secondary antibody goat anti-rabbit IgG H+L Alexa Flour 488 (ab11008).

### 2.12. Enzyme-Linked Immunosorbent Assay (ELISA)

Secreted levels of the pro-inflammatory molecules, interleukin (IL)-6, monocyte chemotactic protein-1 (MCP-1), and regulated on activation normal T cell expressed and secreted (RANTES) were measured by ELISA and normalized to the weight of tissues according to the manufacturer’s instructions (R&D Systems, Minneapolis, MN, USA). The optical density was read at A_450_ with wavelength correction at A_570_ on a Synergy HTX plate reader from BioTek (Winooski, VT, USA).

### 2.13. Growth Factor Assay

Growth factor secretion was measured using the Mouse Growth Factor Array (AAM-GF-3-8) from Ray Biotech (Peachtree Corners, GA, USA). Brain homogenates were used to semi-quantitatively detect the levels of 10 mouse proteins, according to the manufacturer’s instructions. Signal levels were detected by chemiluminescence and visualized using a ChemiDoc imaging system (Bio-Rad). Densitometric analysis was measured using image J (NIH.gov) and intensities normalized to positive control on each membrane.

### 2.14. Gene Array Assay

mRNA was isolated from brain tissues using the miRNeasy Mini Kit (Qiagen; catalog # 217004) and the purity of the RNA was measured on a microplate reader (BioTek). RNA preparations with an O.D. 260/280 nm absorbance ratio of at least 2.0 were used for cDNA synthesis, followed by gene measurements using the Mouse Autophagy RT² Profiler PCR Array (Qiagen). One microgram of RNA was used for the first strand cDNA synthesis using Qiagen’s RT^2^ First Strand Kit (catalog # 330401), as per the supplier’s protocol. A genomic DNA elimination step was performed before reverse transcription. The relative abundance of each mRNA species was assessed using RT^2^ SYBR Green/ROX PCR master mix (Qiagen; catalog # 330520), containing 0.5 µg of RNA aliquoted in equal volumes (25 µL) to each well of the real-time PCR array, using a BioRad CFX96 real-time system (Bio-Rad). The threshold cycle (Ct) of each gene was determined by using BioRad software. The threshold and baseline were set manually, according to the manufacturer’s instructions. Ct data were uploaded into the data analysis template on the manufacturer’s website (http://pcrdataanalysis.sabiosciences.com/pcr/arrayanalysis.php, accessed on 8 April 2022). The relative expression of each gene was calculated using the ΔΔCT method with five housekeeping genes and compared with the expression in control cells. 

### 2.15. Statistical Considerations

For all experiments, data are presented as the mean ± SEM. Tests for significant differences between the groups in experiments regarding the characterization of EVs released by different types of parental cells were performed using a one-way ANOVA with multiple comparisons (Fisher’s pairwise comparisons) using GraphPad Prism, version 5.0 or higher (GraphPad software, San Diego, CA, USA).

## 3. Results

### 3.1. Manufacturing and Characterization of TPP1-Loaded EVs

**Figure 1:**
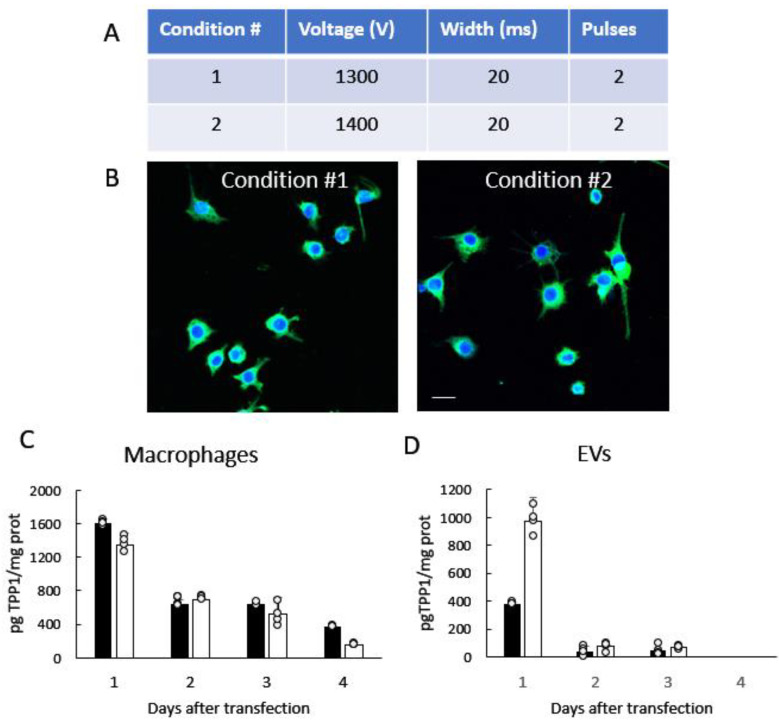
Genetic modification of parent macrophages with TPP1-encoding *p*DNA and TPP1 levels in EVs released by the cells**.** (**A**) Macrophages were transfected with TPP1 *p*DNA tagged with Myc using the Invitrogen Neon Electroporation system with two different electroporation conditions. (**B**) Twenty-four h after the transfection, the expression of the encoded protein Myc-TPP1 was visualized with FITC anti-Myc tag antibody (ab1263, green). Nuclei were stained with DAPI (blue). (**C**,**D**) The kinetics of TPP1 expression in parent macrophages and EVs released by the cells were assessed by ELISA over four days after the transfection with condition #1 (black bars) and condition #2 (white bars). Scale bar: 20 µm. Data were analyzed using two-way ANOVA analysis. Values are means ± SEM.

We demonstrated earlier that immunocyte-derived EVs have a superior ability to reach inflamed brain tissues and deliver their therapeutic payload [26,36,37,38]. Thus, the brain accumulation of macrophage-derived EVs was significantly higher compared to neuron-derived and astrocyte-derived EVs [38]. Based on these findings, macrophages were chosen as a source for the manufacture of EV-TPP1. Specifically, parent cells were transfected with TPP1-encoding plasmid DNA (*p*DNA) under two different electroporation conditions (Figure 1A) using the Invitrogen Neon Electroporation system. To differentiate between TPP1 synthesized de novo in the transfected cells and the endogenous protein, TPP1-encoding *p*DNA with Myc tag was utilized (Supplementary Figure S1). Twenty-four h after transfection, the expression of TPP1 in the parent cells was visualized by confocal microscopy with an antibody to Myc (Figure 1B). Both electroporation conditions resulted in the significant expression of TPP1 in macrophages. As expected, no fluorescence was detected in control cells transfected with sham *p*DNA (Supplementary Figure S2). TPP1-loaded EVs were collected from parent macrophages conditioned media and characterized for size and distribution using the Nanoparticle Tracking Analysis (NTA) and Dynamic Light Scattering (DLS), as described in the Materials and Methods section. According to the NTA data, the average diameter mean of the collected EV-TPP1 was 122.8 ± 13.6 nm and the mode 99.3 ± 3.5 nm. DLS further proved the Z _avg_ was 99.1 ± 5.9 nm. The expression levels of proteins specific for EVs (HSP90, TSG101, and CD63) were confirmed by Western blot analysis, using Wes™ (Supplementary Table S1 and Figure S3). 

Next, the presence of the encoded therapeutic protein TPP1 in the cells and EVs was assessed over four days after the transfection by ELISA. In this experiment, TPP1-encoding *p*DNA without Myc tag was used (Supplementary Figure S4). The highest levels of TPP1 expression in parent macrophages (Figure 1C), as well as in EVs (Figure 1D), were recorded in the first days after transfection. Based on the data, the transfection condition #2, which provided the highest levels of TPP1 in EVs (Figure 1D), was chosen for all further experiments.

### 3.2. Biodistribution of ^64^Cu-Labeled EVs in CLN2 Mice by PET

**Figure 2:**
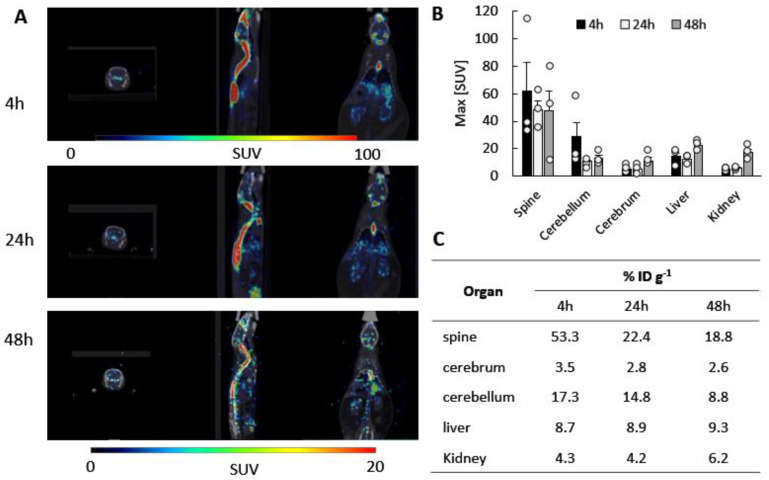
Biodistribution kinetics of ^64^Cu-labeled EVs in CLN2 mice by PET. Radioactively labeled EVs were injected into CLN2 mice (2 mo. of age) through *i.t*. route (2 × 10^10^ particles/50 µL). The animals were imaged by PET over 48 h time period after the injection (**A**). At the endpoint (48 h), mice were sacrificed and perfused, and the radioactivity in the blood and main organs was measured. Panels present (**A**) representative PET images, (**B**) image-derived standardized uptake values SUV max, and (**C**) percentage of the injected dose (%ID/g) quantified for major organs. The calculations confirmed the greatest brain accumulation of ^64^Cu-EVs in spine and brain areas in CLN2 mice. Individual data points shown in Supplementary Table S2. Data were analyzed using two-way ANOVA analysis. Values are means ± SEM.

Successful therapy of BD requires efficient delivery of enzymatically active TPP1 to all organs; however, the most challenging is the transfer to the CNS. Our earlier investigations using Bioluminescence Imaging (IVIS) indicated that the intrathecal (*i.t.*) administration route provided a superior brain accumulation of EVs nanocarriers in CLN2 mice [28]. It is likely that the *i.t.* route allowed bypassing EVs entrapment in peripheral organs and, therefore, provided better brain bioavailability. Herein, we studied the accumulation of macrophage-derived EVs in CLN2 mice administered via *i.t.* injection by PET imaging, a highly sensitive technique that detects pairs of gamma rays emitted indirectly by a positron-emitting radionuclide. For this purpose, EVs were labeled with ^64^Cu, and CLN2 mice (2 mo. old) received a single intrathecal injection of ^64^Cu-EVs (48.0 ± 2.8 µCi/2 × 10^10^ particles/50 µL/mouse, N = 3). The representative images of CLN2 mice injected with ^64^Cu-EVs are presented in Figure 2A. The standardized uptake values, SUV_max_, in the main organs for ^64^Cu-EVs were assessed at 1 h, 24 h, and 48 h post injection (Figure 2B). SUV_max_ was calculated as the tissue uptake level, normalized by the injected radiotracer activity and body mass ((kBq mL^−1^)/(kBq g^−1^)). ^64^Cu-EVs brain levels for each animal are shown in Supplementary Table S2. Importantly, *i.t.* injection yielded the highest levels of ^64^Cu-EVs in the spine and brain areas. Thus, the cerebral SUV_max_ values for ^64^Cu-EVs after *i.t.* injection exceeded those in the main organs, liver, and kidney. The low clearance of the carriers from 24 h to 48 h post injection indicated extended retention of EVs in the brain. 

To analyze the biodistribution of drug carriers, %ID g^−1^ values were calculated for the main organs of interest: the brain, liver, and kidney (Figure 2C). Individual values of the injected dose (%ID/g) for each animal are presented in Supplementary Table S2. The data indicated that the highest ^64^Cu-EVs levels were detected at the 4 h point in the spine and cerebellum. The EVs accumulation levels in the peripheral organs, liver, and spleen were considerably lower, which signifies the superior brain bioavailability for EVs, up to 20.7% of the injected dose/g (including the cerebrum and cerebellum). More than 53% of the injected dose was detected in the spine at the 4 h point (Figure 2C). The same pattern was observed for the later time points. 

### 3.3. Cumulative Effect of Repetitive Administrations of DIR-EVs in CLN2 Mice by Bioluminescence Imaging (IVIS)

The labeling with ^64^Cu does not allow tracing drug carriers for a prolonged time period. On the other hand, labeling EVs with a near-infrared lipophilic fluorescent dye, 1,1′-Dioctadecyl-3,3,3′,3′-tetramethylindo-carbocyanine iodide (DIR), allows detecting EVs over 20 days after a single injection [27]. Therefore, the biodistribution of EVs upon repetitive administrations over several weeks was studied in CLN2 mice with DIR-EVs using IVIS. This regimen was chosen because it reflected more accurate multiple injections used in therapeutic efficacy studies with CLN2 mice. Our investigations revealed that *i.t.* injections provided drug delivery to the brain, while systemic intraperitoneal (*i.p.*) administration led to EVs accumulation in peripheral organs: liver, kidney, spleen, and lungs [27]. Thus, the necessity for delivery of TPP1 to all organs dictates the combination of *i.p.* and *i.t.* injections of EV-TPP1 that should be the most effective and potent therapeutic approach. 

**Figure 3:**
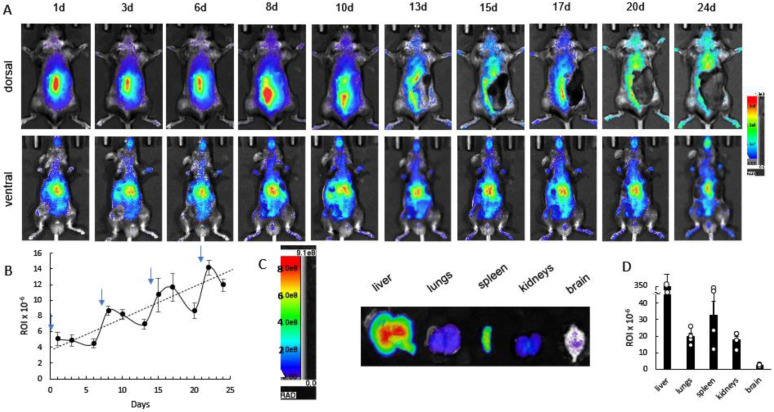
Cumulative effect of repetitive administrations of EVs resulting in buildup of drug nanocarriers. CLN2 mice (2 mo. old) were injected with DIR-labeled EVs through the combination of *i.p.* (6 × 10^11^ particles/200 µL) and *i.t* injections (1.5 × 10^11^ particles/50 µL) once a week and imaged up to 24 days by IVIS. (**A**) Representative images show a buildup of DIR signal in the brain and main organs. (**B**) Quantification of DIR-EVs distribution in the brain at various time points confirmed elevated DIR-EVs signal in the brain. (**C**) Twenty-four days later, the main organs were imaged by IVIS, and (**D**) the fluorescence levels of DIR-EVs signals were quantified by Aura software. Data were analyzed using two-way ANOVA analysis. Values are means ± SEM, *N* = 4. Arrows—days of injections.

Herein, we investigated the biodistribution of EVs in CLN2 mice (2 mo. old, *N* = 4) subjected to weekly administrations via a combinational approach using *i.t.* and *i.p.* injections by IVIS (Figure 3). Dorsal and ventral representative images taken over four weeks revealed a considerable amount of DIR-EVs in the main organs of CLN2 mice (Figure 3A). Importantly, the quantitative analysis of the in vivo images indicated a steady-state increase in the brain accumulation of EVs throughout all time frames of the experiment (Figure 3B). Representative dorsal and ventral images of control mice injected with saline did not show any fluorescence (Supplementary Figure S5). At the endpoint, animals were sacrificed and perfused to eliminate EVs in the blood stream, and the main organs (i.e., liver, lungs, spleen, kidney, and brain) recovered postmortem were imaged by IVIS (Figure 3C). The highest levels of DIR-EVs were detected in the liver and spleen, with a lower amount in the lungs, kidneys, and brain (Figure 3D). The steady increases in EVs accumulation in the brain upon repetitive administrations may provide efficient TPP1 delivery, supporting significant therapeutic effects in CLN2 mice.

### 3.4. Neuroprotective Therapeutic Actions of EV-TPP1 in the Brains of CLN2 Mice

Herein, we confirmed the neuroprotective therapeutic effects of EV-TPP1 administrations in CLN2 animals, when compared to CLN2 animals administered with sham EVs or saline. CLN2 mice (*n* = 10) were treated with EV-TPP1 formulations through a combination of *i.p.* injections (4.5 × 10^10^ particles/150 µL/mouse, every week from day 10) and *i.t.* injections (1.5 × 10^10^ particles/50 µL/mouse, every week from month 1). Wild type (WT) mice and CLN2 mice injected with saline were used as positive (healthy) and negative (sick) controls, respectively. Another control group of CLN2 mice was treated with sham EVs, according to the same schedule and regiments. Three months later, mice were sacrificed and perfused, and the brains were collected and frozen in OCT media, as described in the Materials and Methods section. 

**Figure 4:**
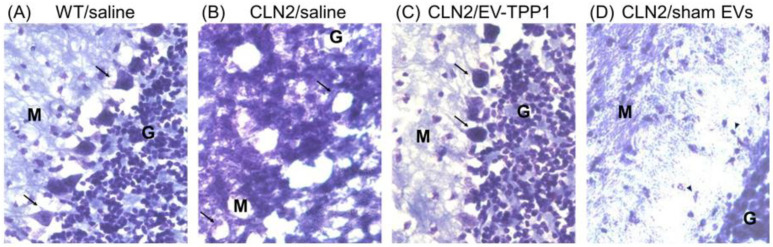
Histological analysis of brain tissues using Nissl staining. Brain sections from healthy (**A**) or CLN2 mice (10 d. old) were treated with saline (**B**), EV-TPP1 (**C**), or sham EVs (**D**) via combination of *i.p.* and *i.t.* injections as described in the Materials and Methods section. Normal Purkinje cells displaced upward into the molecular layer (arrow) detected in brain tissues recovered from healthy control (**A**) and EV-TPP1-treated CLN2 (**C**) mice. Densely populated granule cell layer (G) and the uniform molecular layer (M) in the healthy control (**A**) and EV-TPP1-treated CLN2 (**C**) brain section. Absence of Purkinje cells with large vacuolated molecular layer (M) in saline-treated CLN2 (**B**) and neuronal shrinkage (arrowhead) with sparsely populated molecular layer (M) and granule cell layer (G) in sham-EV-treated CLN2 (**D**) brain sections.

Frozen brains were cut into coronal sections and stained with Nissl staining for the visualizing of neurons in the cerebral cortex (Figure 4). Upon binding to the rough endoplasmic reticulum in neuronal perikarya and dendrites, Nissl staining provides a marker for the physiological state of the neuron [39]. Postmortem brain recovered from WT (healthy) mice treated with saline (Figure 4A) showed a typical morphology of Purkinje neurons (arrows), including the nucleus, cell body, and axon, that appeared normal, with no evidence of neuronal swelling and vacuolation. Densely populated granule cell layer (G) and the uniform molecular layer (M) were noticeable in the healthy control brain sections. On the contrary, the postmortem brain recovered from CLN2 mice treated with saline (Figure 4B) showed noticeable evidence of pathology, with an absence of Purkinje cells, neuronal swelling and vacuolation, and cell loss, were observed (Figure 4B, arrows). In contrast to the saline-treated CLN2 mice, brains recovered from EV-TPP1-treated CLN2 mice showed a normal morphology with no sign of pathology. The Purkinje neurons in the densely populated granule cell layer (arrows) appeared typical (Figure 4C). Finally, postmortem brain recovered from CLN2 mice treated with sham EVs (Figure 4D) showed a healthier tissue morphology, when compared to brains recovered from CLN2 mice treated with saline (Figure 4B), although considerable evidence of neuronal shrinkage (arrowhead) with sparsely populated molecular layer and granule cell layer were observed (Figure 4D). 

**Figure 5:**
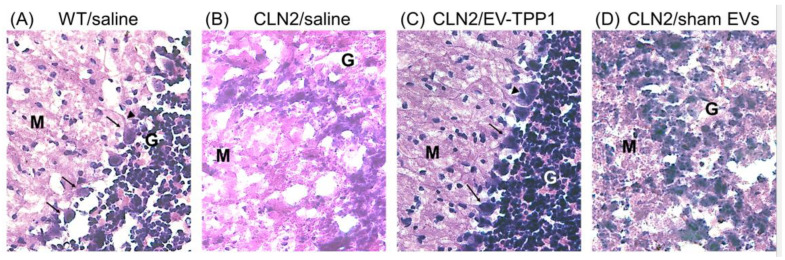
Histological analysis of brain tissues using H&E staining. Brain sections from healthy (**A**) or CLN2 mice (10 d. old) treated with saline (**B**), EV-TPP1 (**C**), or sham EVs (**D**), via combination of *i.p.* and *i.t.* injections as described in the Materials and Methods section. Normal Purkinje cells displaced in the molecular layer (M) (arrow) and cell dendrites (arrows) detected mostly in brain tissues recovered from healthy control (**A**) and EV-TPP1-treated CLN2 (**C**) mice. Densely populated granule cell layer (G) and the uniform molecular layer (M) in the healthy control (**A**) and EV-TPP1-treated CLN2 (**C**) brain section. Hypocellular granule cell layer (G), an absence of Purkinje cells, and a vacuolated and loosely arranged molecular layer (M) in saline-treated CLN2 (**B**) and sham-EV-treated CLN2 (**D**) brain sections.

Another subset of sliced tissues was stained with Hematoxylin and Eosin (H&E) for further detection of morphological changes among the different treatments (Figure 5). Like what was observed with Nissl, tissues recovered from healthy WT (healthy) mice showed a typical morphology (Figure 5A). On the contrary, no clear distinction between the granular (G) and molecular (M) layers were observed, and severe damage was visible in tissues recovered from saline treated CLN2 mice (Figure 5B). Postmortem brain tissues recovered from EV-TPP1-treated CLN2 mice had a well-defined area of nuclei in the granular layer that were stained blue, while the cytoplasm and extracellular matrix showed varying degrees of pink staining (Figure 5C, arrows). Normal Purkinje cells (arrow) including nucleus, cell body and dendrites were detected in the molecular layer (Figure 5C). Lastly, tissues recovered from CLN2 mice treated with sham EVs showed absence of Purkinje cells, with a vacuolated and loosely arranged molecular layer (Figure 5D). 

Overall, postmortem brain tissues recovered from animals treated with EV-TPP1 showed superior tissue integrity, with minor visible signs of aberrant neuronal cell morphology in the cerebellar cortex. This suggests that EV-TPP1 interventions rescued brain morphology by enhancing neuronal survival in CLN2 brain tissues. On the contrary, the microscopic appearance by histology showed considerable signs of tissue damage and loss of Nissl substance in the brain of CLN2 mice treated with saline and sham EVs. 

**Figure 6:**
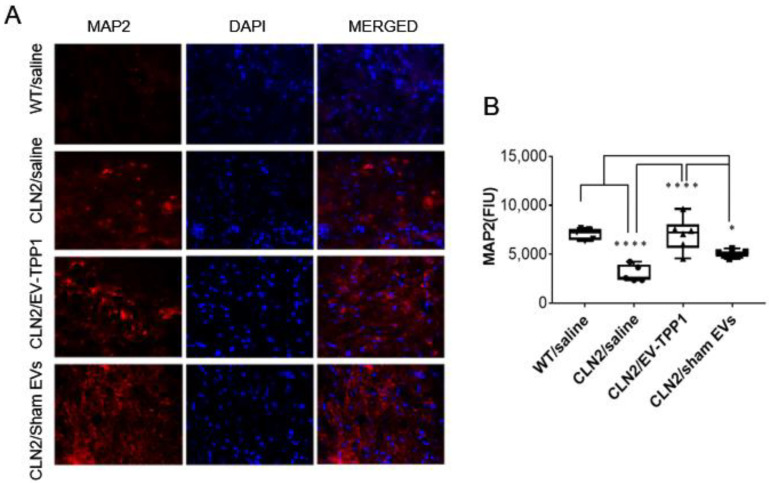
Immunohistochemical analysis of neuroprotective effects by EV-TPP1 in BD mice using neuronal staining. CLN2 mice (10 d. old) were treated with saline, EV-TPP1, sham EVs, or TPP1 alone via combination of *i.p.* and *i.t.* injections as described in the Materials and Methods section. Wild type control mice were injected with saline. Three months later, animals were sacrificed, and sliced brain tissues were stained with MAP2 (red) and DAPI (nuclei). Fluorescent images were taken with a confocal microscope (**A**), and the fluorescence intensity unit (FIU) of MAP2 was quantified (**B**) using the Zen Software (Zeiss). Results are reported as the mean ± SEM of three independent experiments. Data were analyzed using one- or two-way ANOVA analysis followed by Tukey’s multiple comparisons test. Values are means ± SEM, **** *p* < 0.0001; * *p* < 0.05. Scale bar: 20 µm.

To further confirm the neuroprotective action of EV-TPP1, sliced brain tissues were immunohistochemically labeled with the antibody against microtubule-associated protein 2 (MAP2), for the assessment of neuronal health. MAP2 is a neuron-specific protein that directly binds to microtubules in the dendrites of postmitotic neurons and influences microtubule dynamics and microtubule/actin interactions to control neurite outgrowth and synaptic functions. Antibodies to MAP2 are excellent markers for the detection of neuronal cells, their perikarya, and neuronal dendrites. Moreover, given the essential roles the microtubule cytoskeleton plays during mitosis in neurons, a reduced level of MAP2 expression correlates with disruption of the dynamic instability of microtubules, which can lead to mitotic block, cell cycle arrest, and cell death [40]. Fluorescent images illustrated in Figure 6A, were quantified using the Zen Software (Zeiss) (Figure 6B). When compared to brain tissues recovered from saline treated CLN2 mice, the intensity of MAP2 was significantly higher in brain tissues recovered from WT (healthy) mice, and in brain tissues recovered from EV-TPP1 treated CLN2 mice (Figure 6A,B). Interestingly, MAP2 intensity in brain tissues recovered from sham-EVs-treated CLN2 mice was higher than in saline treated CLN2 mice, although significantly lower when compared to the MAP2 intensity in brain tissues recovered from EV-TPP1-treated CLN2 mice. Overall, treatment with EV-TPP1 showed increased neuronal survival and brain integrity, suggesting neuroprotective effects in CLN2 mice. 

### 3.5. Anti-Inflammatory Therapeutic Actions of EV-TPP1 in Brains of CLN2 Mice

**Figure 7:**
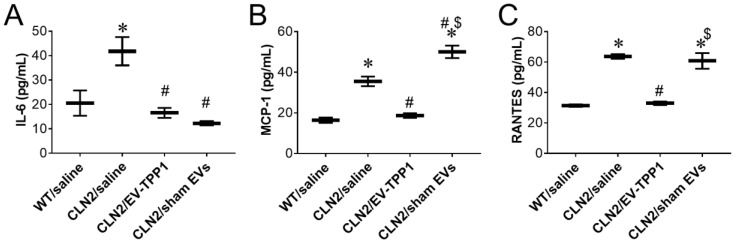
Effect of EV-TPP1 treatments on the levels of inflammatory signals in mouse brain. CLN2 mice (10 d. old) were treated with saline, EV-TPP1, sham EVs, or TPP1 alone via combination of *i.p.* and *i.t.* injections as described in the Materials and Methods section. Wild type control mice were injected with saline. Three months later, animals were sacrificed, and the inflammation state in the brain was assessed by the levels of IL-6 (**A**), MCP-1 (**B**), and RANTES (**C**) using ELISA and normalized to weight of tissues. Results are reported as the mean ± SEM of three independent experiments. Data were analyzed using one- or two-way ANOVA analysis followed by Tukey’s multiple comparisons test. A value of *p* < 0.05 was considered significant. * vs. WT/saline; # vs. CLN2/saline; $ vs. CLN2/EV-TPP1.

Neuroinflammation has been recognized as an important characteristic at the pathological and cellular levels in NCLs [41,42]. On that note, the anti-inflammatory action of the EV-TPP1 formulation in mouse brain tissues was assessed by measuring the secretion of various pro-inflammatory cytokines and chemokines. Brains recovered postmortem were isolated and homogenized in tissue buffer as described previously [43]. The findings showed levels of pro-inflammatory molecules that were about 2.0-fold higher in saline-treated CLN2 brain when compared to healthy WT mice (Figure 7), thus providing evidence of the neuroinflammation associated with BD. Moreover, EV-TPP1-treated CLN2 brain showed a 2.5-fold decrease in the secretion of IL-6 (Figure 7A) and a 1.9-fold decrease in MCP-1 and RANTES (Figure 7B,C) when compared to saline-treated CLN2 brain. In fact, EV-TPP1 attenuated the inflammatory molecules almost to the same levels detected in healthy WT brains (Figure 7A–C). Interestingly, sham-EVs-treated CLN2 brain also showed anti-inflammatory actions by decreasing IL-6 levels (Figure 7A), while no significant changes were measured in the secretion of MCP-1 and RANTES (Figure 7 B,C) when compared to saline-treated CLN2 brain. 

**Figure 8:**
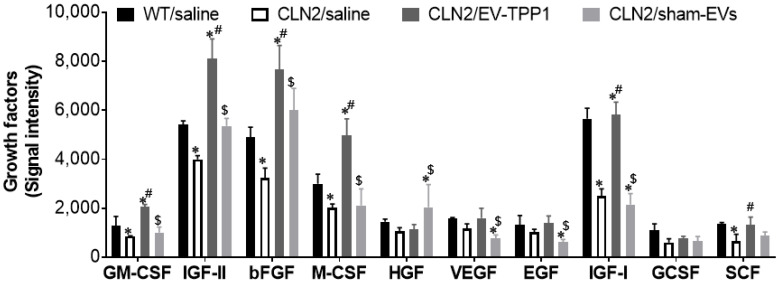
Effect of EV-TPP1 treatment on expression of different growth factors in mouse brain. CLN2 mice (10 d. old) were treated with saline, EV-TPP1, sham EVs, or TPP1 alone via combination of *i.p.* and *i.t.* injections as described in the Materials and Methods section Wild type control mice were injected with saline. Animals were sacrificed 3 months later, brains were harvested, and collected tissue lysates were used to semi quantitatively detect the levels of 10 mouse growth factors, according to manufacturer’s instructions. Signal levels were detected by chemiluminescence, and intensities normalized to positive control on each membrane. The intensity of each protein was measured using Image J software (NIH.gov). Results are reported as the mean ± SEM of three independent experiments. Data were analyzed using one- or two-way ANOVA analysis followed by Tukey’s multiple comparisons test. A value of *p* < 0.05 was considered significant. * vs. WT/saline; # vs. CLN2/saline; $ vs. CLN2/EV-TPP1.

Brain homogenates were used to semi-quantitatively detect the secretion of 10 mouse growth factor proteins (Figure 8) [43]. Levels of granulocyte-macrophage colony-stimulating factor (GM-CSF), insulin-like growth factor-I and II (IGF-I, IGF-II), basic fibroblast growth factor (bFGF), macrophage colony-stimulating factor (M-CSF) and stem cell factor (SCF) were significantly decreased in tissues recovered from saline-treated CLN2 mice (Figure 8, white bars), by 1,4-, 1.4-, 2.3-1.5-1.5-, and 2.0-fold, respectively, when compared to saline-treated WT (healthy) brain tissues (Figure 8, black bars). On the contrary, levels of the above-mentioned growth factors doubled in brain tissues recovered from EV-TPP1-treated CLN2 mice (Figure 8, dark gray bars) when compared to saline-treated CLN2 brain tissues (Figure 8, white bars). Lastly, significant decrease in growth factors were measured in tissues recovered from sham EVs-treated CLN2 mice (Figure 8, light gray bars) when compared to tissues recovered from EV-TPP1-treated CLN2 mice and healthy control mice. Signal levels of different growth factors on separate membrane are represented in Supplementary Figure S6. Overall treatment with EV-TPP1 caused a decrease in the secretion of inflammatory molecules concurrent with an increase in the secretion of several growth factors involved in neuroprotective functions, including synaptic formation, neuronal plasticity, protein synthesis, and autophagy [44,45]. 

### 3.6. Potential Mechanism Mediating the Therapeutic Effects of EV-TPP1 Treatments in Brains of CLN2 Mice

#### 3.6.1. Elimination of Lipofuscin Aggregates in CLN2 Brain

We hypothesized that enzymatically active TPP1 delivered to the brain of CLN2 mice can destroy lipofuscin aggregates that would result in significant neuroprotection. To examine this possible process, histological analysis of CLN2-disease-related neuropathological changes (i.e., storage material accumulation) was performed on different brain regions. Immunostaining for subunit C of mitochondrial ATP synthase (SCMAS), the main protein component of lysosomal storage material, revealed pronounced SCMAS immunoreactivity in all examined brain areas (cerebellum, frontal lobe, motor cortex, and pons). 

**Figure 9:**
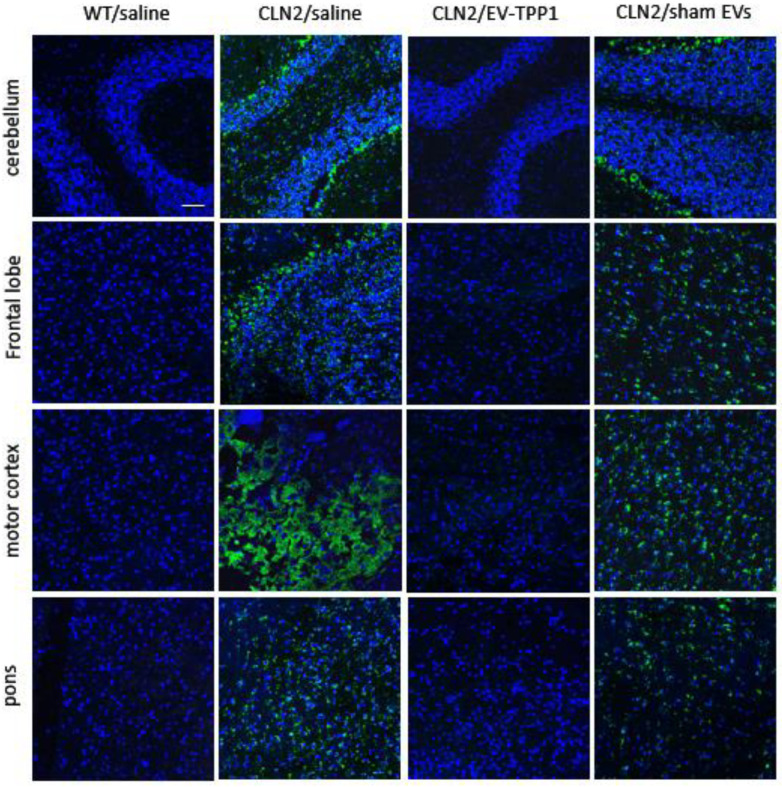
EV-TPP1 treatment resulted in significant elimination of storage accumulation (subunit C of mitochondrial ATP synthase) in the brains of CLN2 mice. CLN2 mice (10 d. old) were treated with saline, EV-TPP1, sham EVs, or TPP1 alone via combination of *i.p.* and *i.t.* injections once a week. Wild type control mice were injected with saline. Three months later, animals were sacrificed, and sliced brain tissues were stained with antibody to subunit C of mitochondrial ATP synthase (A) as described in the Materials and Methods section. Immunohistochemical analysis of subunit C of mitochondrial ATP synthase (SCMAS) revealed a robust treatment effect of EV-TPP1 upon the treatment. This was not seen in sham-EVs-treated CLN2 mice. Scale bar: 50 µm.

For this purpose, CLN2 mice (*N* = 10) were treated with the EV-TPP1 formulation, as described above. Wild type (WT) mice and CLN2 mice injected with saline were used as positive (healthy) and negative (sick) controls, respectively. Another control group of CLN2 mice was treated with sham EVs according to the same schedule and regiments. Three months later, mice were sacrificed and perfused, and brains were collected and examined for storage material by immunohistochemistry, as described in the Materials and Methods section. Saline-injected CLN2 mice in the control group showed a prominent accumulation of cytoplasmic storage material within the lysosomal andendosomal compartments (Figure 9A). In contrast, treatments with EV-TPP1 significantly diminished the accumulation of lysosomal storage bodies, almost to the levels present in WT mice. The quantification of the fluorescent levels of the confocal images confirmed significant decreases in subunit C of mitochondrial ATP synthase in all examined brain areas in CLN2 mice treated with EV-TPP1 (Supplementary Table S3). Of note, treatments with sham EVs caused some decreases in protein storage material, although to a lesser extent compared to EV-TPP1. 

#### 3.6.2. Activation of Autophagy-Related Mechanisms Involved in the Formation of Autophagosome with Possible Sign of Autophagic Flux

**Figure 10:**
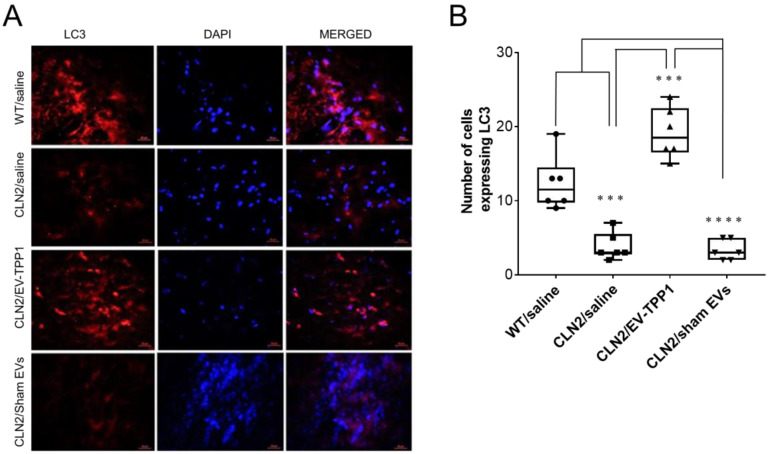
Effect of EV-TPP1 treatment on expression of LC3 in brain of CLN2 mice. CLN2 mice (10 d. old) were treated with saline, EV-TPP1, sham EVs, or TPP1 alone via combination of *i.p.* and *i.t.* injections as described in the Materials and Methods section. Wild type control mice were injected with saline. Three months later, animals were sacrificed, and sliced brain tissues were stained with antibody to LC3 (**A**). Nuclei were visualized with DAPI staining. (**B**). Immunoreactivity was measured using Zen software, and the numbers of cells expressing the protein were plotted in box and whiskers plots using GraphPad Prism. Results are reported as the mean ± SEM of three independent experiments. Data were analyzed using one- or two-way ANOVA analysis followed by Tukey’s multiple comparisons test. **** *p* < 0.0001; *** *p* < 0.001. Scale bar: 20 µm.

There is growing evidence across a variety of non-mammalian and mammalian model systems that clearly supports an effect of *CLN* gene mutations on autophagy, suggesting that autophagic dysregulation contributes to NCL pathology [46]. To this end, we investigated whether EV-TPP1 treatments affected the expression of microtubule-associted protein 1A/1B-light chain 3 (LC3) protein in CLN2 brain (Figure 10).

Sliced brain tissues were immunohistochemically labeled with the antibody against the microtubule-associated protein light chain 3 (LC3) for monitoring autophagy and autophagy-related processes [47]. Representative fluorescence images showed significant decrease in the immunoreactivity of LC3 in brain tissues recovered from CLN2 mice treated with saline when compared to healthy control mice (Figure 10A,B). A significant increase in the expression of LC3 was detected in brain slides obtained from CLN2 mice treated with EV-TPP1. In fact, LC3 expression levels in EV-TPP1-treated CLN2 brain was about twofold higher than that in saline-treated CLN2 brain (Figure 10A,B), while treatment with sham EVs did not affect LC3 immunoreactivity in brain tissues recovered from CLN2 mice. 

**Figure 11:**
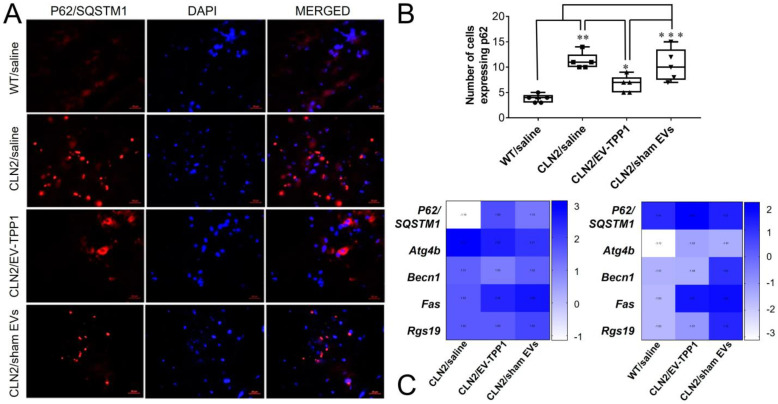
Effect of EV-TPP1 treatment on expression of P62 in brain of CLN2 mice. CLN2 mice (10 d. old) were treated with saline, EV-TPP1, sham EVs, or TPP1 alone via combination of *i.p.* and *i.t.* injections as described in the Materials and Methods section. Wild type control mice were injected with saline. Three months later, animals were sacrificed, and sliced brain tissues were stained with antibody against SQSTM1/P62 (**A**). Nuclei were visualized with DAPI staining. SQSTM1/P62 immunoreactivity was measured using Zen software, and the numbers of cells expressing the protein were plotted in box and whiskers plots using GraphPad Prism (**B**). Relative mRNA expression of the indicated genes was measured in brain tissues using autophagy PCR arrays, and the numbers are plotted in a heat map as fold change from CLN2/saline (left panel) and as fold change from WT/saline (right panel) using GraphPad Prism (**C**). Data were analyzed using one- or two-way ANOVA analysis followed by Tukey’s multiple comparisons test. *** *p* < 0.0001; ** *p* < 0.005; * *p* < 0.05. Scale bar: 20 µm.

Since we recognized the expression of LC3 in CLN2 brain as a marker for activation of autophagy, next, we assessed whether activation leads to maturation and degradation of autophagosome. Postmortem brain tissues were immunolabeled with antibody against SQSTM1/P62 for monitoring maturation and autophagic flux [47]. Representative fluorescence images showed increase expression levels of SQSTM1/P62 immunoreactivity in tissue slides recovered from saline treated CLN2 mice (Figure 11A,B), indicative of a blockade in the maturation of the autophagy pathway potentially due to a lack of fusion between the lysosome and autophagosome. The number of cells expressing SQSTM1/P62 in tissues recovered from saline-treated CLN2 mice was about 1.8-fold higher than in tissues recovered from EV-TPP1-treated CLN2 mice. Interestingly, levels of SQSTM1/P62 immunoreactivity in CLN2 brain treated with sham EVs were significantly higher when compared to brain tissues recovered from EV-TPP1-treated CLN2 mice (Figure 11A,B), suggesting that the TPP1 enzyme and not EV alone, is required for lysosomal fusion. 

Lastly, the autophagy-related genes associated with CLN2 brain tissues were measured with an RT-Profiler gene array, as described in the Materials and Methods section. The array examined 84 different mRNA expression profiles of common genes involved in various stages of the autophagy pathway, and the most affected ones are presented in Figure 11C. As shown in the heat map, SQSTM1/P62 mRNA was expressed at a higher level in brain homogenates recovered from EV-TPP1-treated CLN2 mice when compared to tissues recovered from healthy control group (left panel) and saline treated CLN2 mice (right panel) (Figure 11C), suggesting that the reduction of the protein detected in this group (Figure 10A,B) was not a result of reduced de novo synthesis of the *SQSTM1/P62* gene at transcriptional levels. Overall, it appears that EV-TPP1 causes an upregulation in SQSTM1/P62 mRNA that leads to the translation of the protein and degradation by lysosome–autophagosome fusion. Although speculative, we postulate that the de novo synthesis of SQSTM1/P62 at the transcriptional (mRNA) level perhaps restored the impaired lysosome–autophagy fusion in the CLN2 brain after treatment with EV-TPP1. This could account for the improvement in neuronal health (detected by the increased immunoreactivity of MAP2, increased secretion of growth factors, and reduced secretion of cytokines) and the overall integrity of the brain morphology (detected by the appearance of healthy Purkinje cells, densely populated granule cell layer and the uniform populated molecular layer). Ongoing studies in the lab are testing our assumption using additional mouse models of BD. 

## 4. Discussion

There have been several studies investigating the use of EVs as drug delivery vehicles. Several groups report successful loading and delivery of therapeutic proteins to the brain by means of EVs for drug delivery in animal models, including neurodegenerative disorders [23,26,36,48,49,50,51,52,53], lysosomal storage diseases [27,28], and multiple sclerosis [54]. These studies have shown promising results and suggested that EVs may be an effective means of transporting drugs to the brain in these disorders. For example, brain-derived neurotrophic factor (BDNF) was delivered successfully using EVs to the brain parenchyma in healthy mice and at even greater amounts to the inflamed brains [23]. In another study, a potent antioxidant catalase was loaded into exosomes ex vivo and produced significant neuroprotective effects using in vitro and in vivo models of Parkinson’s disease [49]. Yet, one of the main challenges of EV-based therapeutics is the efficient loading of these natural nanocarriers with therapeutic proteins without altering the structure and content of EVs membranes. In the present study, we demonstrated a successful loading of EVs with the therapeutic enzyme TPP1 via genetic modification of parent macrophages with TPP1-encopding *p*DNA. We adjusted experimental conditions to achieve efficient transfection of macrophages and confirmed TPP1 expression in parent cells and EVs with ELISA. 

Another hurdle for these therapeutic interventions, especially in the case of neurodegenerative disorders, is the efficient targeting of EVs with incorporated drug to the disease site. To this point, we suggest utilizing immunocytes as a source for EVs production that target inflamed tissues. We reported earlier a significant increase in the accumulation levels of EVs released by inflammatory response cells, monocytes, and macrophages in the inflamed brain tissues [23,36]. As reported earlier by us, macrophage derived EVs (mEVs) were found to have a superior ability to accumulate in the inflamed mouse brain, compared to astrocyte-derived EVs (aEVs) or neuron-derived EVs (nEVs), although in vitro nEVs were taken up by neurons in greater quantity [38]. We showed that the unidirectional brain influx rate (Ki) and initial volume of brain distribution (Vi) of EVs in the brain-inflamed mice were respectively three- and twofold higher than those in the healthy mice. Consistent with the increased brain influx rate, the brain accumulation of macrophage-derived EVs in the inflamed brain at 10 min was 5.8-fold higher than that in the healthy mice [23]. We uncovered the mechanism of mEVs superior accumulation in the inflamed brain, and these mEVs showed the highest levels of tetraspanins and integrins compared with nEVs and aEVs, which allows higher adhesion and targeting to the inflamed tissues by mEVs. The targeting effect was also supported by the interaction between the integrin lymphocyte function-associated antigen 1 (LFA-1) overexpressed on the mEVs surface and intercellular adhesion molecule 1 (ICAM-1) on the inflamed endothelial cells of brain microcapillaries [23]. 

Herein, we utilized macrophage-derived EVs and established optimal schedule and administration routes for treatment with EV-TPP1 formulations in CLN2 mice. Based on our previous data, we used a combination of two administration routes, *i.t.* and *i.p.* injections. The first one provided proficient delivery of the enzyme to the brain, and the last supplied TPP1 to the main peripheral organs including the liver, spleen, and kidneys. Subsequently, we studied the biodistribution of fluorescently labeled EVs in the main organs of CLN2 mice upon multiple administrations using imaging in living animals (IVIS). The studies revealed the steady-state increase of EVs accumulation upon multiple injections, which suggested sufficient delivery of the incorporated TPP1 to the brain. This resulted in significant restoration of neuronal structure and tissue integrity in the brain of CLN2 mice treated with EV-TPP1, as was showed by histological and immunohistochemical analyses. Thus, noticeable healthy morphology in tissue structure and high integrity of neurons was detected in the brain of CLN2 mice treated with EV-TPP1 when comparted to CLN2 mice treated with saline. Specifically, EV-TPP1 treatments resulted in rescued astrocytes morphology and enhanced neuronal survival in CLN2 brain tissues. Furthermore, a significant decrease in pro-inflammatory molecules (MCP-1 and RANTES), along with an increase in the levels of different growth factors (GM-CSF, bFGF, M-CSF and IGF), were detected in CLN2 mice treated with EV-TPP1 when compared to CLN2 mice treated with saline. These findings are important considering that both G-CSF and GM-CSF were shown to have a protective function in neurons [45], while IGF-1 was known to exhibit neuroprotective functions, including synaptic formation, neuronal plasticity, protein synthesis, and autophagy [44]. Furthermore, hematopoietic trophic cytokine, such as GM-CSF, was shown to inhibit the neurodegenerative effects of paraquat in a Parkinson’s disease mouse model [55]. IGFs stimulates DNA synthesis, cell proliferation, neurite outgrowth, axonal growth, and myelination and enhances the secretion of various neurotransmitters. According to others, in many neurodegenerative diseases, decreases in IGF-1 signals [56] and lack of IGF-1 in the brain may induce apoptosis [57,58]. In some neurodegenerative diseases, M-CSF and microglia were shown to be highly beneficial in clearing amyloid, preventing its toxicity to neurons, and improving cognitive impairment. Importantly, sham EVs did not cause significant therapeutic effects in CLN2 brain.

Regarding the mechanism of EV-TPP1 therapeutic effects, our initial hypothesis was that EVs would facilitate targeted delivery of TPP1, especially to the brain, and protect neurons against the appearance of lysosome aggregates. In fact, neuronal accumulation of autofluorescent lipopigments resembling ceroid and lipofuscin, as well as mitochondrial ATP synthase subunit C, has been found in CLN patients [59,60]. As such, we investigated whether treatments with EV-TPP1 resulted in clearance of these aggregates. As expected, accumulation of EV-TPP1 in the brain caused desegregation of protein aggregates and protection of neurons. These effects were significantly greater in mice treated with EV-TPP1 formulations compared to CLN2 mice treated with sham EVs. This suggests that delivery of TPP1 by means of EVs or/and maybe delivery of TPP1-encoding DNA and de novo synthesis in the CLN2 mouse brain are responsible for these therapeutic effects. To clarify the last point about de novo synthesis of TPP1, we demonstrated earlier that along with the encoded enzyme, EVs released by pre-transfected macrophages contain TPP1-encoding DNA [27], suggesting that along with the enzyme transfer, EV-TPP1 released by transfected macrophages may also deliver TPP1-encoding DNA and accomplish gene delivery to the brain of CLN2 mice. This would result in the transfection of brain tissues and de novo synthesis of TPP1 promotes neuroprotection. 

Another hypothesis is based on the note that accumulation of aggregates due to incapable enzymes in BD patients resulted in the disruption of the autophagic pathway, further producing “collateral damage” in the clearance of protein aggregates. Interestingly, along with BD, this process was reported for different LSDs, including Pompe, Cystinosis, Danon, and Gaucher diseases [30]. As such, autophagy plays a crucial role in the maintenance of lysosomal function in these disorders. Increasing numbers of studies have linked a defective autophagy process to the neuronal ceroid lipofuscinoses [29,61]. The most used techniques include the inhibition of lysosomal function (i.e., by chloroquine) and evaluation of the level of autophagy substrates, such as ubiquitinated proteins and SQSTM1/P62 [62]. LC3 is attached on the cytosolic side of the autophagosome and functions as a receptor for the scaffolding protein SQSTM1/P62, which directs protein cargo for autophagic degradation or flux [63,64,65]. Failure to increase LC3-II following chloroquine treatment and elevated levels of SQSTM1/P62 indicate an impairment of the flux. On the other hand, a decrease in the expression levels of SQSTM1/P62 is indicative of autophagic flux in the autophagy–lysosomal pathway and clearance of intracellular aggregates. In addition to the development of novel methods of delivery of the therapeutic enzyme to the brain, there is ongoing research into the potential use of autophagy-inducing agents as a therapeutic approach for LSDs. While these agents have shown promise in animal models, more research is needed to determine their effectiveness and safety in humans. 

Herein we focused on these two essential proteins (LC3 and SQSTM1/P62) in the autophagy pathway. Of most importance is the notion that EV-TPP1 activated the autophagy pathway, which may have started a secondary process that improved the delivery of aggregates to lysosomes and their clearance. Our findings show that EV-TPP1 induced upregulation of the autophagosome marker LC3 in CLN2 mouse brain. Interestingly, CLN2 mice treated with saline or sham EVs showed a low level of LC3 immunoreactivity, when compared to EV-TPP1-treated CLN2 brain, indicating that autophagy was not being induced. At the same time, only brains administered with EV-TPP1 showed a decrease in the marker of autophagy flux, SQSTM1/p62, suggesting that the neuronal injury detected with CLN2 may be partially attributed to a blockade of autophagy maturation or flux. Interestingly, mRNA analysis revealed upregulation of the *SQSTM1/P62* gene in the CLN2 brain after the EV-TPP1 treatments, suggesting that the decreased levels in protein expression was not due to lack of de novo synthesis gene expression.

## 5. Conclusions

We developed a novel biomimetic drug delivery system for TPP1 based on macrophage derived EVs as drug delivery vehicles. Multiple facets of medicine can be improved with the development of EV-derived drug delivery systems, including tumor treatments, cancer treatments, vaccines, and more. Based on our data and gene array data, we further speculate that the CLN2 brain, upon receiving the EV-TPP1 formulation, not only induces autophagy but is able to restore lysosomal degradation. This, in turn, could be a potential mechanism by which neurons are restored. Enhanced lysosomal degradation can lead to reduced inflammation in the brain and throughout the body. At least two different processes may be supportive of EV-TPP1 treatment: (i) delivery of active TPP1 and TPP1-encoding *p*DNA and (ii) activation of the autophagy process in the brain of CLN2 mice.

Overall, the role of autophagy in LSDs is significant and continues to be an active area of research. While EVs show promise as a tool for the treatment of neurodegenerative disorders, further studies are needed to fully understand their potential and to identify any potential drawbacks or challenges.

## Data Availability

Data supporting reported results can be found at The Neuroscience Multi-omic Archive.

## Supplementary Materials

The following supporting information can be downloaded at: https://www.mdpi.com/article/10.3390/cells12111497/s1, Figure S1: Plasmid map of TPP1-encoding pDNA with Myc tag; Figure S2: Confocal image of control macrophages. The cells were transfected by electroporation in the absence of TPP1-pDNA with Myc tag and seeded onto slide. 24 h later, the cells were treated with FITC anti-Myc tag antibody (ab1263) and imaged by confocal microscopy. Nuclei were stained with DAPI (blue). No fluorescence indicating absence of significant background was recorded. Scale bar = 20 µm; Figure S3: Characterization of EV-TPP1 by Western Blot analysis. Expression levels of EV-specific proteins in EVs (1) and parent cells (2) as well as β-actin was assessed by Wes. In most cases EVs show higher levels of examined proteins compared to cells. β-actin levels were greater in cells compared to EVs; Figure S4: TPP1-encoding pDNA production, and verification. A: plasmid map for TPP1 production, B: Giga Prep verification of TPP1-pDNA; Figure S5: Images of untreated control mice by IVIS. Representative dorsal and ventral images of control mice injected with saline did not show any fluorescence; Figure S6: Effect of EV-TPP1 treatment on expression of different growth factors in mouse brain. Imaging of the membranes showing both positive and negative data; Table S1: Primary antibodies used for Simple Western Blot; Table S2: %ID/g in the main organs of animals injected with 64Cu-EVs.
