# Supplementary material for: Extracellular Vesicles Released by Genetically Modified Macrophages Activate Autophagy and Produce Potent Neuroprotection in Mouse Model of Lysosomal Storage Disorder, Batten Disease
Source: Cells. 2023 May 29;12(11):1497. doi: 10.3390/cells12111497 (PMC10252192; doi:10.3390/cells12111497)
Supplement: Supplementary file 1 [file cells-12-01497-s001.zip › cells-2362447-supplementary.pdf]

Supporting Information

Macrophage Derived Extracellular Vesicles Loaded with a Therapeutic Enzyme Activate Autophagy and Produce Potent Neuroprotection in a Mouse Model of Lysosomal Storage Disorder, Batten Disease

Nazira El-Hage[1], Matthew J. Haney[2,3], Yuling Zhao[2,3], Myosotys Rodriguez[1], Mori Liu[3], Carson J. Swain[3], and Elena V. Batrakova[2,3,*]

[1] Florida International University myrodrig@fiu.edu (M.R.); nelhage@fiu.edu (N. E-H.)

[2] Center for Nanotechnology in Drug Delivery; University of North Carolina at Chapel Hill, Chapel Hill, NC 27599, USA; yulingz@email.unc.edu (Y.Z.); mjhaney@email.unc.edu (M.J.H.); batrakov@ad.unc.edu (E.V.B.)

[3] Eshelman School of Pharmacy, University of North Carolina at Chapel Hill, Chapel Hill, North Carolina, USA; jfallon@email.unc.edu (J.K.F.); camiarzt@live.unc.edu (C.J.A.); pcs@email.unc.edu (P.C.S.); matthew loop@unc.edu (M.S.L.)

*Correspondence should be addressed to E.V.B. (batrakov@ad.unc.edu)

UNC Eshelman School of Pharmacy, University of North Carolina at Chapel Hill, Chapel Hill, NC 27599-7362, Phone: 919-537-3712, Email: batrakov@ad.unc.edu

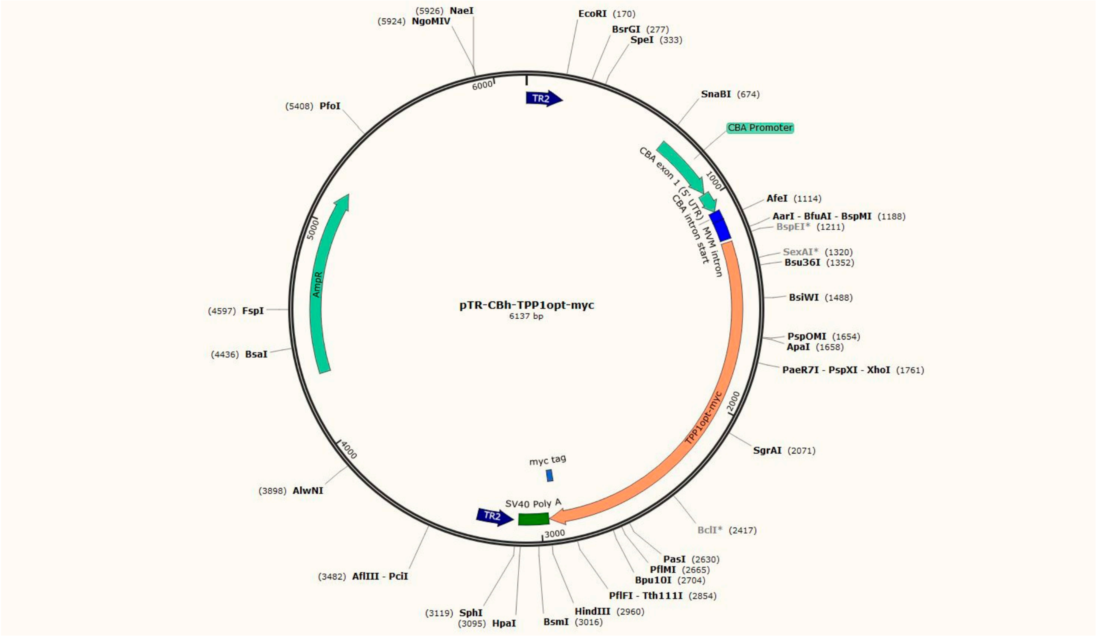

Supplementary Figure S1. Plasmid map of TPP1-encoding *p*DNA with Myc tag.

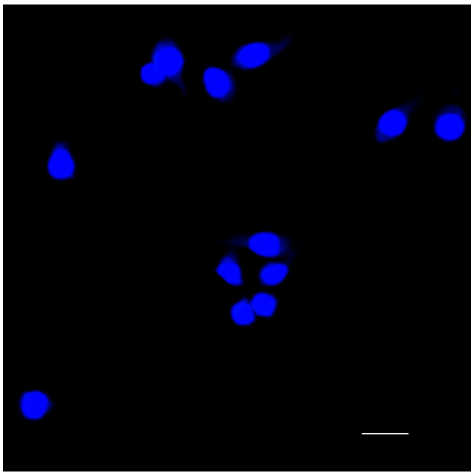

Supplementary Figure S2. Confocal image of control macrophages. The cells were transfected by electroporation in the absence of TPP1-*p*DNA with Myc tag and seeded onto slide. 24 h later, the cells were treated with FITC anti-Myc tag antibody (ab1263) and imaged by confocal microscopy. Nuclei were stained with DAPI (blue). No fluorescence indicating absence of significant background was recorded. Scale bar = 20 µm.

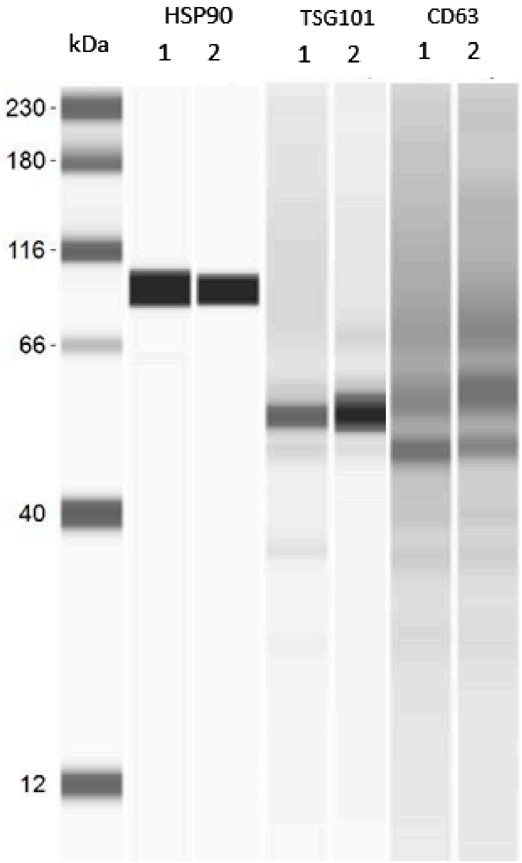

Protein	Band MW, kDa	Area x 10^4	
		EVs	Cells
HSP90	92 - 94	203.7	151.6
TSG101	53 - 54	14.0	41.9
CD63	25 - 60	20.8	19.7

Supplementary Figure S3. Characterization of EV-TPP1 by Western Blot analysis. Expression levels of EV-specific proteins in EVs (**1**) and parent cells (**2**) were assessed by Wes. In most cases EVs show higher levels of examined proteins compared to cells.

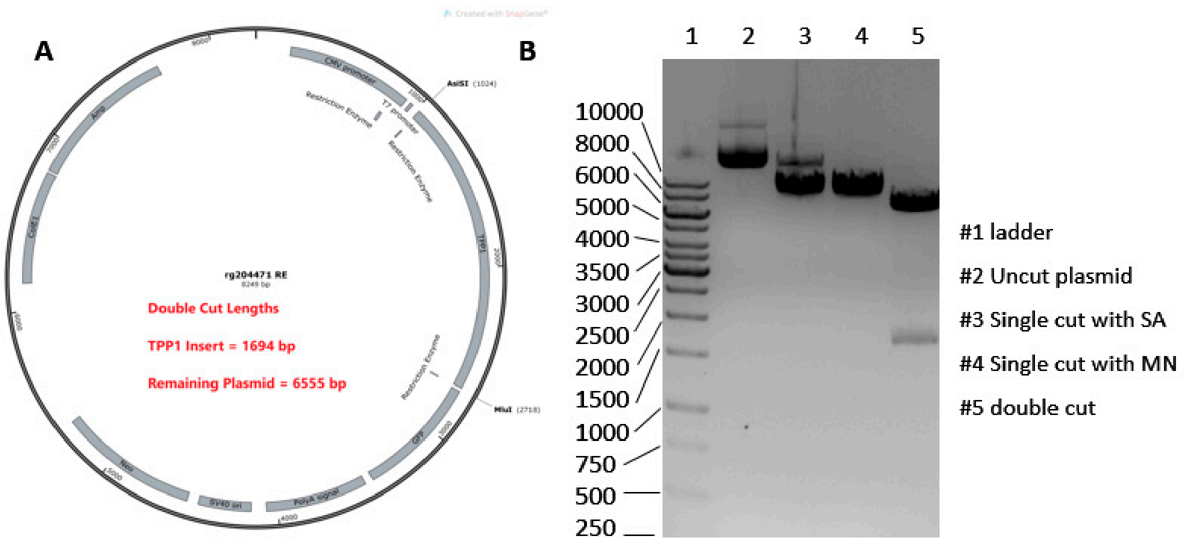

Supplementary Figure S4. TPP1-encoding *p*DNA production, and verification. A: plasmid map for TPP1 production, **B**: Giga Prep verification of TPP1-*p*DNA.

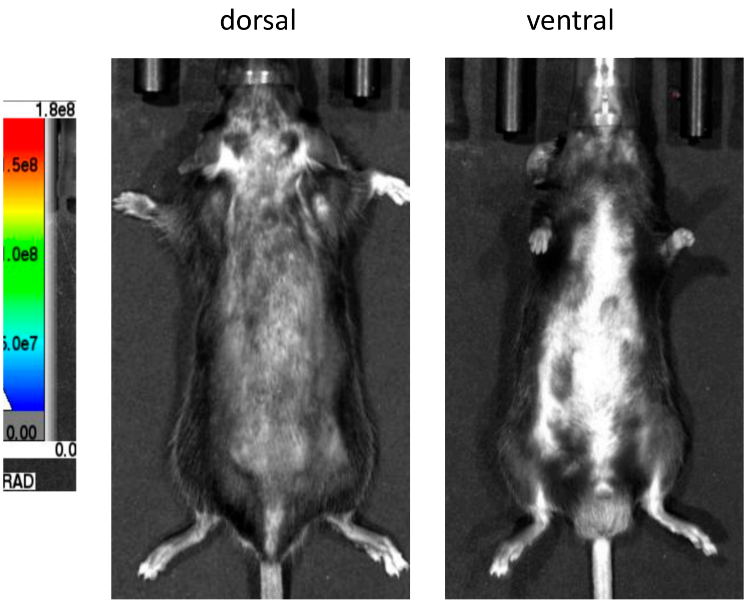

Supplementary Figure S5. Images of untreated control mice by IVIS. Representative dorsal and ventral images of control mice injected with saline did not show any fluorescence.

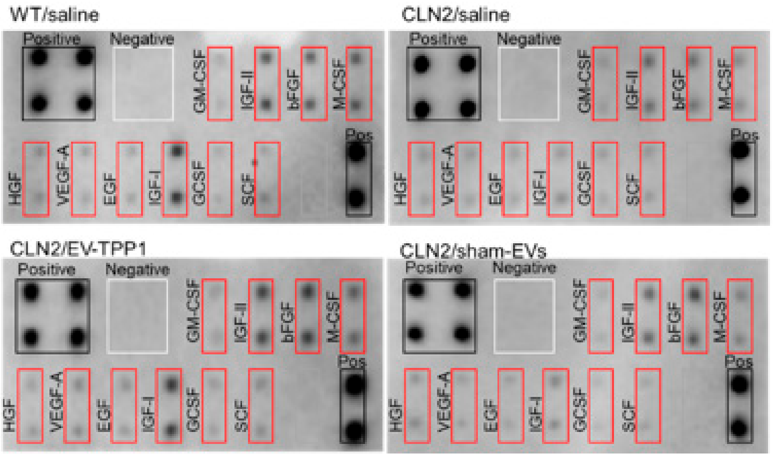

Supplementary Figure S6. Effect of EV-TPP1 treatment on expression of different growth factors in mouse brain. Imaging of the membranes showing both positive and negative data.

Supplementary Table S1. Primary antibodies used for Simple Western Blot

Antibody	Manufacturer	ID	Stock concentration mg/ml	Dilution factor	Protein concentration mg/ml
CD63	Novus	NBP2-67425	1.000	100	40
HSP90 beta	Novus	NBP2-67395	1.000	50	200
TSG101	Novus	NBP2-67884	1.000	500	40
β Actin	Abcam	ab213262	0.500	50	200

Supplementary Table S2. %ID/g in the main organs of animals injected with ^{64}Cu-EVs

Animal	Organ	%ID/g		
		**1h**	**24h**	**48h**
Animal #1	Spine	53.33	22.39	18.77
	Cerebrum	3.48	2.84	2.58
	Cerebellum	17.29	14.82	8.84
	Liver	8.68	8.92	9.25
	Kidney	4.27	4.21	6.18
Animal #2	Spine	25.72	15.66	7.46
	Cerebrum	1.87	1.3	1.2
	Cerebellum	10.64	7.48	6.48
	Liver	30.91	26.45	15.34
	Kidney	6.72	7.14	5.38
Animal #3	Spine	23.36	18.29	11.07
	Cerebrum	2.10	1.83	1.55
	Cerebellum	9.47	6.29	3.37
	Liver	23.26	15.16	15.43
	Kidney	4.56	5.47	4.94

Supplementary Table S3. Quantification of the brain slides of CLN2 mice and WT control animals treated with different EV-based formulations for the presence of subunit c of mitochondrial ATP synthase (SCMAS)*

**Area/treatment**	**WT/saline (FIU)**	**CLN2/saline (FIU)**	**CLN2/EV-TPP1 (FIU)**	**CLN2/sham EVs (FIU)**
Cerebellum	193.0 ± 6.9	967.7 ± 32.7	147.5 ± 4.8 (***)	373.3 ± 23.0
Frontal lobe	304.2 ± 40.3	1004.2 ± 458.2	145.5 ± 7.6 (**)	585.2 ± 37.5
Motor cortex	281.1 ± 34.5	1262.1 ± 263.7	159.9 ± 6.4 (****)	467.2 ± 36.0
Pons	165.3 ± 18.7	661.7 ± 45.5	141.4 ± 6.7 (*****)	463.2 ± 71.5

*Data were analyzed using one or two-way ANOVA analysis followed by Tukey's multiple comparisons test. Values are means ± SEM. $N = 8$, ****P < 0.00001; ***P < 0.0001; **P < 0.005; *P < 0.05, compared to CLN2/saline.
